# Case report: Metagenomics next-generation sequencing in the diagnosis of septic shock due to *Fusobacterium necrophorum* in a 6-year-old child

**DOI:** 10.3389/fcimb.2024.1236630

**Published:** 2024-02-16

**Authors:** Haiyang Zhang, Zhongqiang Liu, Yuanlin Guan, Deyuan Li, Hanmin Liu, Lingying Ruan

**Affiliations:** ^1^ Department of Pediatric Intensive Care Unit, West China Second University Hospital, Sichuan University, Chengdu, China; ^2^ Key Laboratory of Birth Defects and Related Diseases of Women and Children (Sichuan University), Ministry of Education, Chengdu, China; ^3^ Depertment of Bioinformation, Hugobiotech Co., Ltd., Beijing, China; ^4^ Department of Pediatrics, West China Second University Hospital, Sichuan University, Chengdu, China

**Keywords:** *Fusobacterium necrophorum*, sepsis, children, metagenomics next-generation sequencing, case report

## Abstract

*Fusobacterium necrophorum* (*F. necrophorum*) infection is rare in pediatrics. In addition, the detection time of *F. necrophorum* by blood culture is long, and the positive rate is low. Infection with *F. necrophorum* bacilli usually follows rapid disease progression, resulting in high mortality. In previous reports of *F. necrophorum*-related cases, the most dangerous moment of the disease occurred after the appearance of Lemierre’s syndrome. We report an atypical case of a 6-year-old female patient who developed septic shock within 24 h of admission due to *F. necrophorum* infection in the absence of Lemierre’s syndrome. *F. necrophorum* was identified in a blood sample by metagenomics next-generation sequencing (mNGS) but not by standard blood culture. The patient was finally cured and discharged after receiving timely and effective targeted anti-infection treatment. In the present case study, it was observed that the heightened virulence and invasiveness of *F. necrophorum* contribute significantly to its role as a primary pathogen in pediatric septic shock. This can precipitate hemodynamic instability and multiple organ failure, even in the absence of Lemierre’s syndrome. The use of mNGS can deeply and rapidly identify infectious pathogens, guide the use of targeted antibiotics, and greatly improve the survival rate of patients.

## Introduction

*Fusobacterium necrophorum* (*F. necrophorum*) is an obligate anaerobic, gram-negative rod bacterium commonly located in the oropharyngeal, genitourinary, and intestinal tracts. *F. necrophorum* can colonize the human body for a long time as a benign, non-pathogenic bacteria. However, in recent years, there have been a few reports that *F. necrophorum* can transform into community-acquired invasive bacteria, leading to serious infections. It can cause either localized abscesses and throat infections or systemic, life-threatening disease (Almohaya et al., 2020). Lemierre’s syndrome is a rare but potentially fatal complication caused by infection with *F. necrophorum*, which can be defined as bacteremia, internal jugular vein (IJV) thrombophlebitis, and metastatic septic emboli secondary to acute pharyngeal infections (Lee et al., 2020; Carius et al., 2022). The annual incidence rate of Lemierre’s syndrome is relatively low, estimated at approximately one case per million individuals in the general population (Riordan, 2007; Sheehan et al., 2019; Sacco et al., 2019). Typically, previously healthy young adults suffer more from this disease. The incidence is disproportionately concentrated in adolescent populations aged 16–24 years (Almohaya et al., 2020; Carius et al., 2022; Sacco et al., 2019). The mortality rates of this disease reported in the pre-antibiotic era reached 90%, whereas currently, mortality rates range from 5% to 9% (Almohaya et al., 2020; Riordan, 2007; Nygren and Holm, 2020). Currently, advancements in imaging techniques facilitate the prompt identification of Lemierre’s syndrome in clinical practice, thereby enabling timely therapeutic intervention. However, we have found in clinical practice that the infection of *F. necrophorum* in young children is often fatal in a short period of time because of a rapidly progressing inflammatory storm and does not wait for Lemierre’s syndrome to develop. Conventional blood culture is time-consuming and has a very low positive rate, which delays the diagnosis of *F. necrophorum* and timely targeted drug use. Therefore, more rapid and accurate methods are needed to identify *F. necrophorum*, guide early medication, and prevent the spread of infection.

In this case report, we describe a critical incident involving a 6-year-old female patient who developed fatal septic shock, predominantly attributed to a severe infection dominated by *F. necrophorum*. The pathogens were swiftly identified in the patient’s blood through the application of metagenomic next-generation sequencing (mNGS). This patient was eventually cured by targeted anti-infective therapy.

## Case presentation

A 6-year-old female child was admitted to the pediatric intensive care unit (PICU) of West China Second University in December 2022. One day before admission, the patient’s main clinical symptom was hyperpyrexia (>39°C), accompanied by headache, neck pain, sore throat, dysphagia, occasional non-projectile vomiting, and anorexia. Later, those symptoms worsened with new manifestations, including hoarse voice, shortness of breath, mouth breathing, purple lips, accelerated heart rate, and disturbance of consciousness. After an initial emergency visit, she was quickly admitted to the PICU. The girl’s physical development was normal. She had no history of recurrent respiratory infections or other major illnesses since birth. She was vaccinated against COVID-19 and the flu a year ago. Furthermore, she had no history of prior hospitalizations or surgeries. None of her family members had reported an immunodeficiency or an autoimmune disorder. There was no history of pet ownership in her family.

**Figure 1:**
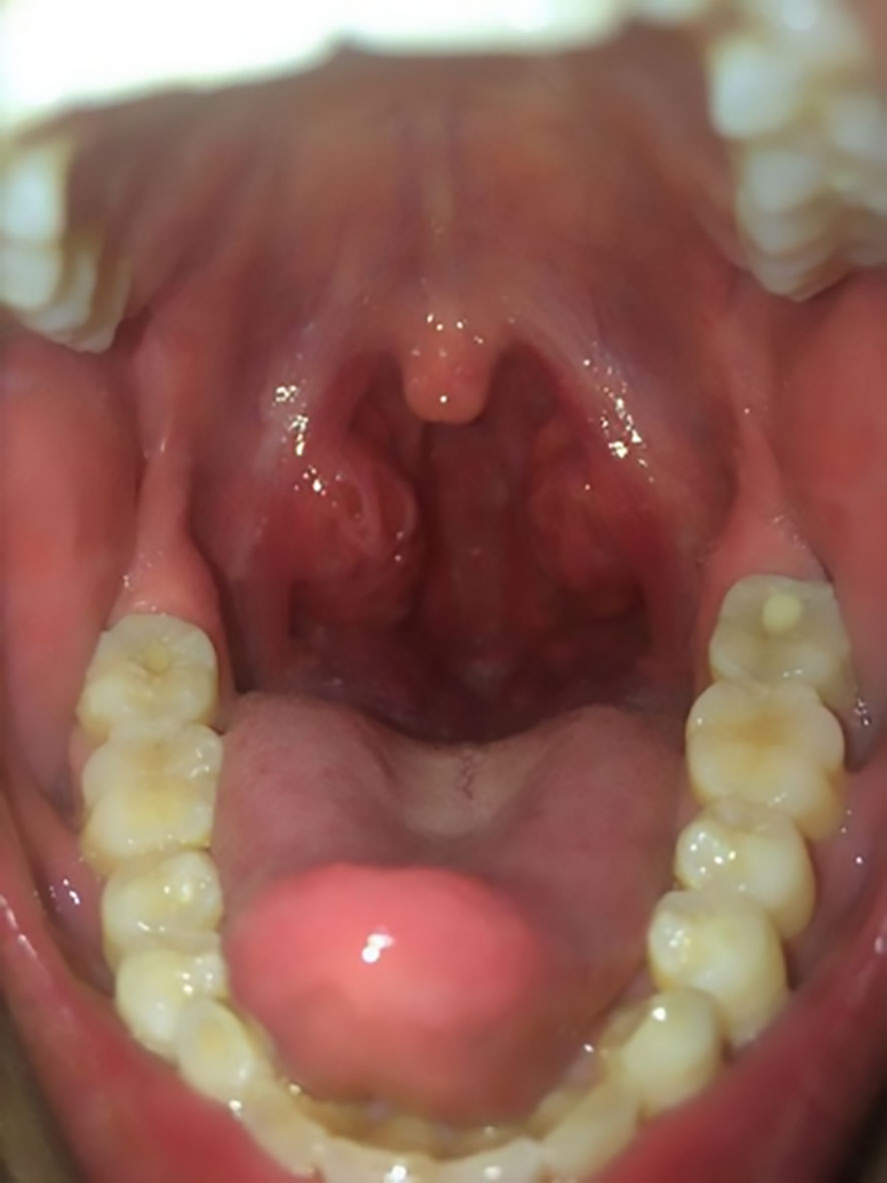
The patient’s oropharynx showed a markedly swollen bilateral tonsil with white, purulent exudates.

**Figure 2:**
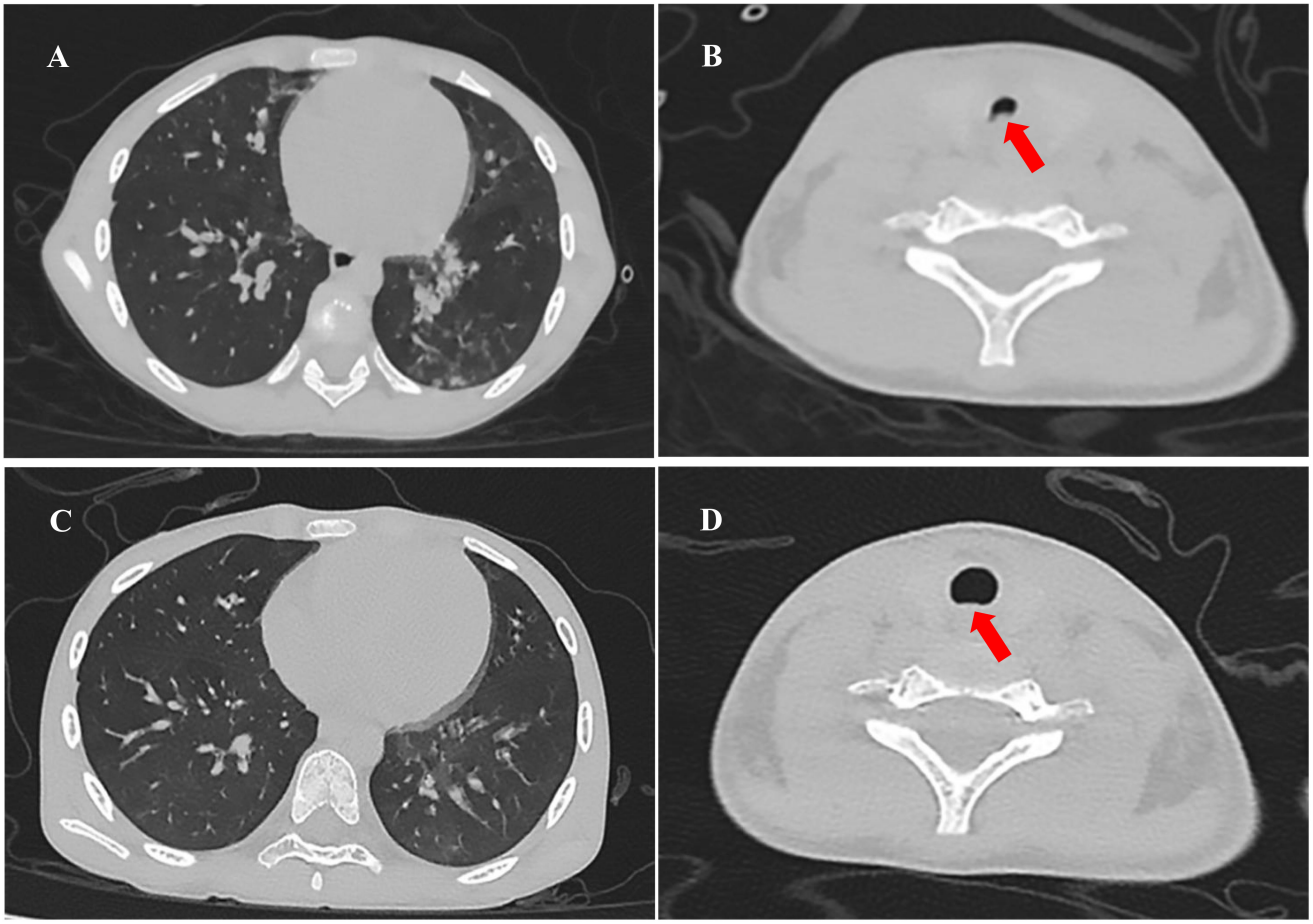
Chest CT in the clinical course of the patient: **(A)** Multiple small patchy hyperdense shadows and nodular shadows in both lungs (the day of admission). **(B)** The laryngeal–pharyngeal area was slightly narrow (red arrow, the day of admission). **(C)** Patchy hyperdense shadows were absorbed, and nodule shadows were significantly reduced (the 14th day of admission). **(D)** No evidence of internal jugular vein thrombophlebitis or abscess, the morphology of the laryngeal pharyngeal area returned to normal (red arrow, the 14th day of admission).

This patient’s vital signs at admission included an increased body temperature (38.8°C), heart rate of 174 beats per minute, respiratory rate of 68 breaths per minute, and blood pressure of 91/52 mmHg. She has severe cyanosis, dyspnea, and oxygen saturation of 75% without oxygen. The Glasgow Coma score was 9 (E3M5V1). The oropharynx showed a markedly swollen bilateral tonsil with small purulent exudates (**Figure 1**). Three concave signs were positive. Inspiratory laryngeal stridor was detected on auscultation. Coarse rales were heard in both lungs. Laboratory investigations yielded the following findings: blood gas analysis indicative of type I respiratory failure; white blood cell count (WBC) at 5.43 × 10^9^/L (reference range: 4.0 × 10^9^/L–12.0 × 10^9^/L) with a predominance of neutrophils (87.3% neutrophils and 8.4% lymphocytes). The hemoglobin (Hb) level measured 101 g/L (reference range: 120 g/L–160 g/L), and the platelet count (PLT) was 123 × 10^9^/L (reference range: 100–300 × 10^9^/L). The C-reactive protein (CRP) concentration was elevated to 29.15 mg/L (0 mg/L–6 mg/L), and that of procalcitonin was 29.52 ng/mL (<0.05 ng/mL). The troponin level was 0.55 ng/mL (more than 10 times higher than the normal value). Coagulation function was significantly abnormal: prothrombin time (PT) was 17.1 s, activated partial thromboplastin time (APTT) was 37.7 s, the international normalized ratio (INR) was 1.57, the D-dimer level was 3.13 mg/L, and the fibrinogen degradation product (FDP) level was 20.74 µg/mL (<5 µg/mL). The antistreptolysin O test (ASO) showed negative results. We collected the patient’s cerebrospinal fluid (CSF) by lumbar puncture (5 mL–10 mL). The counting of nucleated cells, cell classification, protein qualitative test, glucose quantification, chloride quantification, protein quantification, lactate dehydrogenase quantification, and culture of the CSF were negative. Chest CT results showed multiple small patchy hyperdense shadows and nodular shadows in both lungs (**Figure 2A**), and the laryngeal pharyngeal area was slightly narrow (**Figure 2B**). An abdominal CT revealed no liver abscesses or intestinal abnormalities. A head CT showed no abnormalities. There were no abnormalities on cervical vascular ultrasound or abdominal ultrasound.

According to the above clinical symptoms and test parameters, the patient was preliminarily diagnosed with sepsis, severe pneumonia, acute laryngeal obstruction, myocardial damage, and coagulation disorders. She was immediately given a trachea cannula and subsequently treated with invasive mechanical ventilation. Meanwhile, she was empirically given ceftriaxone intravenous drip (50 mg/kg for 1 time, iv drip) combined with vancomycin (15 mg/kg q6h for 2 days, iv drip) for anti-infection, infusion of fresh frozen plasma, gamma globulin (1 g/kg qd for 2 days, iv drip) therapy, and other symptomatic treatments.

**Figure 3:**
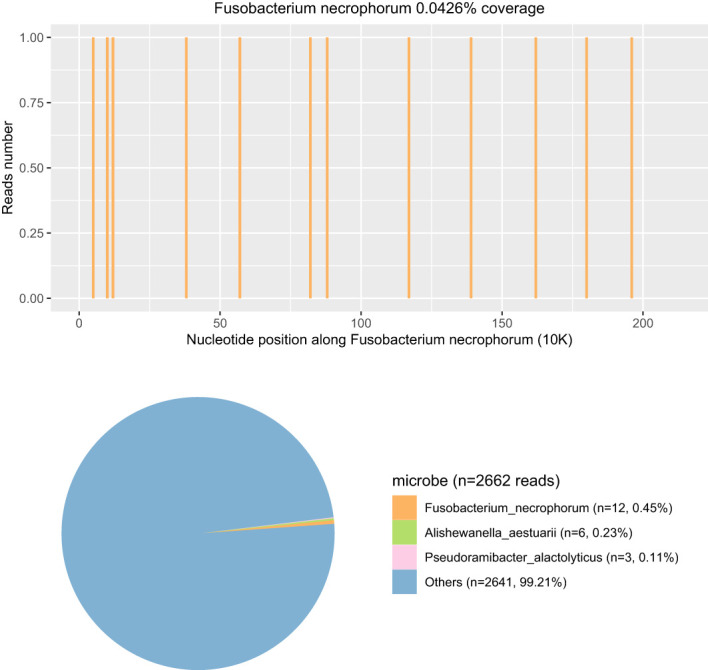
The coverage and abundance of *F. necrophorum* detected by mNGS using blood on the third day of admission. In the Others section, the total number of sequences was 2,642 reads and the percent was 99.21%, including *Prevotella*, *Acinetobacter*, *Stenotrophomonas*, and *Neisseria* (the human reads were removed).

Within 24 h of admission to the PICU, the blood pressure decreased to 56/27 mmHg, and sinus tachycardia and cardiac auscultation showed low heart sounds with diastolic gallop rhythm. The capillary refill time (CRT) of the extremities was 4 s. Blood gas analysis suggested metabolic acidosis with hyperlactacidemia (6 mmol/L). Echocardiography indicated a decrease in left ventricular systolic function (EF 55%, FS 22%) with no evidence of endocarditis. The electrocardiogram showed sinus rhythm and incomplete right bundle branch block. Fluid resuscitation with 0.9% normal saline was performed, and vasoactive drugs were continuously pumped through the central vein to maintain hemodynamic stability: noradrenaline (0.05 μg/kg/min–0.1 μg/kg/min, continuous intravenous pumping) and dobutamine (5 μg/kg/min–10 μg/kg/min, continuous intravenous pumping). Based on the above performance and the relevant authoritative international clinical guidelines, the diagnosis was revised to septic shock and septic-associated cardiomyopathy (Singer et al., 2016). We switched from ceftriaxone to meropenem (40 mg/kg q8h for 14 days, iv drip) for anti-infective treatment and added phosphocreatine infusion for myocardial protection. On the second day of admission, reexamination of the laboratory blood report showed that CRP increased to 116.66 mg/L and procalcitonin increased to 56.30 ng/mL. As the coagulation function test suggested a hypercoagulable state, we gave the patient anticoagulation treatment with low-molecular-weight heparin (75 iu/kg q12h for 12 days, subcutaneous injection). The nucleic acid test by PCR of multiple respiratory pathogens, including the human influenza virus, rhinovirus, adenovirus, para influenza virus, metapneumovirus, SARS-CoV-2, *Mycoplasma pneumoniae*, and *Chlamydia pneumoniae*, showed negative results. On the third day of admission, Pathogen Capture Engine (PACE) seq mNGS (Hugo Biotech, Beijing, China) of blood detected *F. necrophorum* with 12 specific sequences and a relative abundance of 1.47%. Meanwhile, a variety of microbes were detected by mNGS in the blood sample (**Figure 3**). The sepsis and associated diseased condition in this case were likely attributable to a microbial consortium, composed of multiple microorganisms that cooperated with *F. necrophorum*. Among these, *F. necrophorum* was identified as the principal pathogenic microorganism responsible for the sepsis in the patient. The patient was referred with a diagnosis of sepsis with a bloodstream infection. We changed the antibiotic regimen to metronidazole (7.5 mg/kg q8h for 12 days, iv drip) and meropenem to suppress *F. necrophorum* and other pathogens.

**Figure 4:**
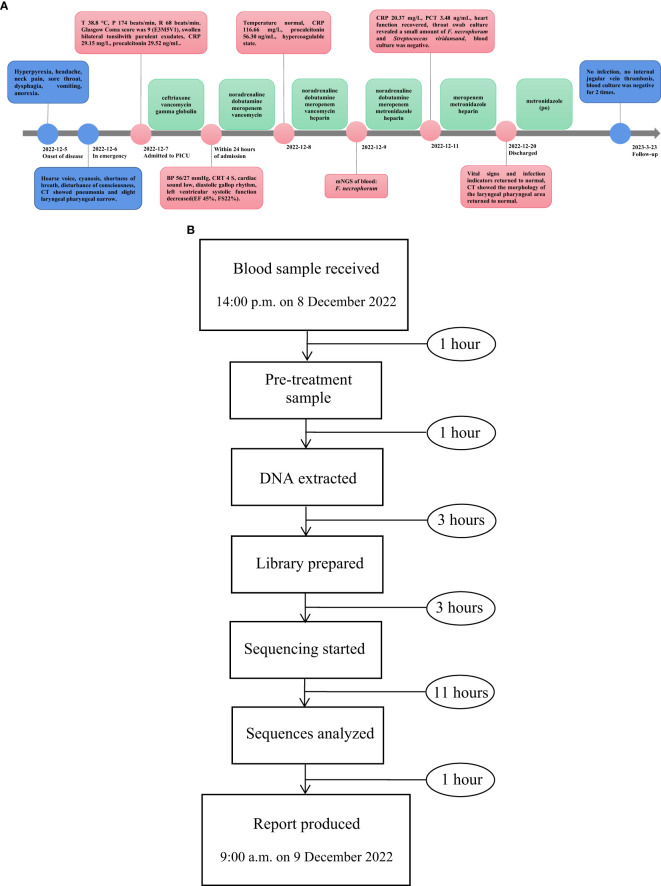
**(A)** The timeline of disease progression and treatment. **(B)** The flowchart of the mNGS process.

On the fifth day of admission, throat swab culture revealed a positive result: a small amount of *F. necrophorum* and *Streptococcus viridans*. Throat swabs were collected on the first day of admission (required and anaerobic bacteria; the culture lasted for 5 days). Matrix-assisted laser desorption/ionization-time of flight mass spectrometry (MALDI-TOF MS; bioMérieux, France) was used to identify the cultured microbes. The patient switched to high-flow nasal cannula oxygen therapy as her spontaneous breathing had returned and laryngeal obstruction had resolved. Her heart function recovered (EF 63%, FS 33%), and vasoactive agents were no longer required to maintain blood pressure. Her temperature remained normal for 3 days, and blood tests showed that inflammatory indicators decreased (CRP 20.37 mg/L, PCT 3.48 ng/mL); coagulation function improved significantly. We performed two blood cultures on the first and third days of admission. In each blood culture collection, blood samples of the left peripheral vein and the right central vein (femoral vein) were collected for blood culture at the same time; that is, two vials of blood culture from different parts were collected at the same time. All blood samples were cultured for 7 days, and both aerobic and anaerobic cultures were performed. On the 10th day, the patient’s two blood cultures showed negative results, respectively. Following a 14-day stay in the PICU, there was a notable improvement in the patient’s condition: dependency on supplemental oxygen ceased, vital signs and infection markers normalized, and chest CT scan revealed absorption of previously observed patchy hyperdense shadows and significant reduction in nodule shadows (**Figure 2C**). Additionally, there was no evidence of internal jugular vein thrombophlebitis or abscess formation. The morphology of the laryngeal pharyngeal area returned to normal (**Figure 2D**). Then, she was discharged for outpatient follow-up for 3 months. Sequential oral metronidazole was continued at discharge (7.5 mg/kg tid for 14 days, po). During the follow-up, there were no signs of infection such as high fever, angina, and dyspnea. There were no neurological or respiratory complications. The inflammatory indicators of WBC, CRP, PCT, and echocardiography were normal. Bilateral cervical ultrasound showed no internal jugular vein thrombosis, and blood cultures were negative twice. The timeline of disease progression and treatment is shown in **Figure 4A**.

## System review

As of 20/05/2022, we have searched the literature of the recent decade in PubMed, Web of Science, Springer Link, and Medline databases with the keywords “*Fusobacterium necrophorum*” and “sepsis”. Patients aged above 18 years were excluded from the study. Finally, a total of 12 articles were selected, and all the clinical data were collected and analyzed, including baseline data, clinical symptoms, means of detection, complications, treatment, and outcome. The characteristics of reported cases are shown in **Table 1** (Sheehan et al., 2019; Gama et al., 2020; Tamura et al., 2020; El Chebib et al., 2020; Repper et al., 2020; Monroe and Amlie-Lefond, 2018; Dal Bo et al., 2019; Whittle et al., 2018; Rana et al., 2017; Sinatra and Alander, 2017; He et al., 2015; Creemers-Schild et al., 2014).

**Table 1:** Published data on patients with *F. necrophorum* sepsis in children under 18 years of age.

No.	Age (year) /sex	Symptom	Medical history	Diagnostic methods	Days from onset to diagnosis	Complications	Treatment	Outcome	References
1	1.5/M	Fever, neck abscess	No	Pyogenic fluids bacterial culture	28	Neck abscess	Triple antibiotic therapy (flucloxacillin, clindamycin, and gentamicin) for 8 days, and co-amoxiclav for 7 days, underwent incision, and drainage of the neck abscess.	Recovery	(Sheehan et al., 2019)
2	17/F	Fever, sore throat, malaise	Recurrent tonsillitis	Blood culture, imaging findings	13	Thrombosis of the left IJV, pulmonary septic embolism.	Amoxicillin and clavulanate potassium + metronidazole for 4 weeks, anticoagulation therapy for 6 weeks.	Recovery	(Gama et al., 2020)
3	17/F	Fever, abdominal pain	No	Blood and abscess cultures	6	Shock, right ovarian abscess.	Laparotomy, drainage, and right adnexectomy, underwent continuous hemodiafiltration, meropenem+vanconmycin+clindamycin for 5 days, cefmetazole for 14 days.	Recovery	(Tamura et al., 2020)
4	15/F	Fever, cough, shortness of breath	No	Blood culture	10	Necrotizing pneumonia, pleural effusion, lung function decline.	Piperacillin tazobactam for 21 days.	Condition improved	(El Chebib et al., 2020)
5	15/F	Fever, sore throat, neck stiffness, headache	No	Blood culture	9	Pulmonary septic embolism, thrombosis of bilateral IJV.	Meropenem for more than 10 days and amoxicillin clavulanate for 4 weeks, anticoagulation therapy for 6 weeks.	Recovery	(Repper et al., 2020)
6	17/M	Cough, sore throat	No	Blood culture	30	Septic emboli to the lungs, kidneys, and spleen, left jugular and dural venous sinus thrombosis, and a carotid space abscess.	CBCT-guided aspiration of abscess in the carotid space, broad-spectrum intravenous antibiotic for more than 4 months.	Condition improved	(Monroe and Amlie-Lefond, 2018)
7	16/M	Fever	No	Blood culture	–	Thrombosis of the left IJV	Prolonged antibiotic therapy and anticoagulation therapy for 4 weeks.	Recovery	(Dal Bo et al., 2019)
8	16/M	Diarrhea, vomiting, abdominal pain	No	Blood culture	11	Right IJV thrombosis, right parapharyngeal abscess, pulmonary abscesses, pleural effusion	VATS pleurodesis procedure, benzylpenicillin and metronidazole for 6 weeks, amoxicillin and metronidazole 2 weeks, anticoagulation therapy for more than 8 weeks.	Recovery	(Whittle et al., 2018)
9	16/M	Sore throat, fever, shortness of breath	No	Blood culture	15	Pulmonary consolidation, pleural effusion	Metronidazole for 10 days.	Recovery	(Rana et al., 2017)
10	15/M	Fever, headache, neck pain	No	Blood culture	7	Epidural abscess spanning T2-L3 level with extension into the psoas and the paraspinal musculature, pulmonary septic embolism.	Hemilaminectomy decompression, meropenem for 6 weeks.	Recovery	(Sinatra and Alander, 2017)
11	17/M	Sore throat, neck stiffness	No	Blood culture	10	Shock, extensive vein thrombosis, vertebral artery dissection, and thrombosis.	Meropenem and metronidazole for more than 6 months, anticoagulation therapy for 6 months.	Condition improved	(He et al., 2015)
12	14/M	Headache, fever, vomiting, malaise, dizziness	No	Blood and nasal swab culture	7	paranasal sinus abscess, intraorbital abscess, right frontal subdural abscess.	Craniotomy+abscess incision drainage, ceftriaxone and metronidazole for 5 days, meropenem and metronidazole for 4 days, penicillin for 7 weeks.	Recovery	(Creemers-Schild et al., 2014)

M, male; F, female; IJV, internal jugular vein; ARDS, acute respiratory distress syndrome; CBCT, cone beam computed tomography; VATS, assisted thoracoscopic surgery.

## Sample collection and nucleic acid extraction

Enough whole blood (children, 2 mL–4 mL) was collected in Cell-Free DNA BCT Streck and then stored or shipped between 6 °C and 35 °C to perform mNGS detection immediately. The DNA was extracted and purified from 200 μL plasma according to the manufacturer’s instructions of QIAamp DNA Micro Kit (50) #56304. DNA concentration and quality were checked through Qubit and agarose gel electrophoresis.

## Library generation and sequencing

The DNA was subjected to library construction through an end-repair method. The DNA libraries were constructed using QIAseq™ Ultralow Input Library Kit. The concentration and quality of libraries were checked using Qubit and agarose gel electrophoresis. Qualified libraries with different barcode labelings were pooled together and then sequenced on an Illumina NextSeq platform. Qualified DNA libraries with different barcode tags were pooled and then sequenced using the Illumina NextSeq 550 sequencing platform (Illumina, San Diego, USA) and a SE75bp sequencing strategy. Shotgun metagenomic sequencing was used in this case.

## Bioinformation pipeline

After obtaining the sequencing data, high-quality data were generated after filtering out adapter, low-quality, low-complexity, and shorter reads. Next, human reads were removed by mapping reads to the human reference genome using SNAP software. The remaining data were aligned to the microbial genome database using Burrows–Wheeler alignment. The database collected microbial genomes from NCBI. It contains more than 20,000 microorganisms, including 11,910 bacteria, 7,103 viruses, 1,046 fungi, and 305 parasites. Finally, the microbial composition of the sample was obtained. In this case, 14 reads of *F. necrophorum* were detected by mNGS, of which 12 were mapped to *F. necrophorum* and 2 reads were mapped to *Fusobacterium* at the genus level. The identity of detected reads to the reference sequences ranged from 98% to 100%. The average read length was 75bp. The flow chart of the mNGS process is shown in **Figure 4B**.

## Positivity criteria

Sterile deionized water was used as the no template control (NTC) and synthesized fragments with known quantities as a positive control (PC). NTC and PC were included in each wet lab procedure and bioinformatics analysis as quality control steps. The positivity criteria of mNGS were as follows: Viruses with non-overlapping reads mapping three distinct genomic regions were reported as positive. For bacteria and fungi (*Mycobacteria* and *Cryptococcus* were excluded), a positive mNGS result was given when its coverage ranked among the top 10 of the same kind of microbes and was absent in the NTC or when its ratio of reads per million (RPM) between sample and NTC (RPM_sample_/RPM_NTC_) > 10 if RPM_NTC_≠0. For *M. tuberculosis* and *Cryptococcus*, a positive mNGS result was considered when at least one unique read was mapped to species level and absent in NTC or RPM_sample_/RPM_NTC_ > 5 when RPM_NTC_≠0.

## Discussion

*F. necrophorum* constitutes a significant representative species within the *Fusobacterium* genus. It accounts for a minute fraction (less than 1%) of human infections caused by non-spore-forming anaerobic bacteria, and the literature only contains a few hundred case reports; there are even rarer cases in pediatrics (Carius et al., 2022). However, it is arguably unparalleled among non-spore-forming anaerobes due to its exceptionally strong association with clinically distinctive and severe septicemic infections that are referred to as Lemierre’s syndrome (Almohaya et al., 2020; Lee et al., 2020; Carius et al., 2022; Sheehan et al., 2019). In the pre-antibiotic era, this syndrome had a high mortality rate of up to 90%, with patients succumbing to a fatal course within 7–15 days. However, early and targeted use of antibiotics can greatly reduce the death rate from the disease (Almohaya et al., 2020; Lee et al., 2020; Carius et al., 2022; Riordan, 2007; Sacco et al., 2019; Nygren and Holm, 2020).

Leukocytotoxin, endotoxin, hemolysin, and hemagglutinin are the bacterial virulence factors of *F. necrophorum*, which have strong biological activity (Brennan and Garrett, 2019). In addition to its role in inducing platelet aggregation, which results in venous thrombosis and septic thrombophlebitis, this entity also plays a pivotal role in the development of metastatic septic embolization and the formation of characteristic abscesses (Riordan, 2007; Sheehan et al., 2019; Sacco et al., 2019). The primary infection site of *F. necrophorum* is commonly in the oropharynx, from which the infection spreads to surrounding tissues. Once *F. necrophorum* invades the internal jugular vein and enters the bloodstream, bacteremia can cause platelet aggregation and thrombosis, facilitating the further spread of infection and the development of metastatic septic embolism and metastatic abscess (Lee et al., 2020; Sheehan et al., 2019; Sacco et al., 2019; Tay and Vasoo, 2022). The phenomenon that distant bacterial transmission can directly impair prognosis is considered to be the main cause of severe infection in adult patients (Sacco et al., 2019). However, in addition to its propensity to cause abscesses or thrombosis, *F. necrophorum* carries strong adhesins and pili, resulting in its remarkable ability to adhere to gram-negative bacteria and gram-positive plaque microorganisms in biofilms, making it a highly invasive opportunistic infectious agent. For pediatric patients, as the immune function was not complete, *F. necrophorum* was more direct and rapid-invading (Brennan and Garrett, 2019). Due to the high virulence and invasiveness of the bacteria, the body would directly respond to severe sepsis, leading to hemodynamic failure and multiple-organ failure prior to internal jugular vein thrombosis and septic embolism (Sheehan et al., 2019; Tamura et al., 2020; El Chebib et al., 2020; Habib et al., 2019). In this case, in the absence of suppurative thrombophlebitis of the internal jugular vein, the *F. necrophorum* infection progressed very rapidly, leading to septic shock within 24 h and causing life-threatening failure of vital organs, including respiratory failure and heart failure. It is suggested that the pathophysiological changes and development of the disease in children infected with *F. necrophorum* may be different from those in adults. In the pathogenesis of sepsis, *F. necrophorum* exhibits synergistic interactions with other bacterial species, contributing to the disease’s progression. This synergism is primarily facilitated by the bacterium’s adhesin (FadA) and fimbriae, which enable adherence to host cells and soft tissues. Moreover, *F. necrophorum* can form extensive biofilms with various bacteria, such as *Escherichia coli* and *Streptococcus hemolyticus*, enhancing their proliferation. This co-aggregation promotes the invasion of typically non-invasive bacterial species, exacerbating sepsis and leading to multiple-organ dysfunction (Lee et al., 2020; Brennan and Garrett, 2019; Groeger et al., 2022). Additionally, *F. necrophorum*’s impact extends to disrupting the host’s normal microbiota, such as the intestinal and oral flora. This disruption results in an imbalance of the microbiota, further modulating the host immune response, thereby aggravating the infection and facilitating its spread (Hou et al., 2022). In this case, we considered that the septic shock of the child was due to a complex bacterial infection dominated by *F. necrophorum*.

The etiological identification of the disease often relies on bacterial culture. Blood culture is important diagnostic evidence. However, it is difficult to make a timely and accurate diagnosis of *F. necrophorum* infection by blood culture because of its obligate anaerobic condition, harsh culture conditions, long culture time (mean 6–8 days), and low positive rate (Riordan, 2007; Sacco et al., 2019; Sato et al., 2021). Through a review of the literature in **Table 1**, we found that only 12 cases of *F. necrophorum* infection have been successfully confirmed in children. Only one patient was under 12 years of age. The detection methods were all conventional bacterial culture methods, and the time from onset to diagnosis of the pathogen was 6–28 days. PCR technology is limited to the detection of anaerobic bacteria. At present, PCR is not routinely used to identify *F. necrophorum*. Sepsis in children caused by *F. necrophorum* may occur within 3 days after infection. Earlier recognition and treatment are imperative because of the possible extrapharyngeal sequalae and high mortality in the absence of antimicrobial treatment. Therefore, there is an urgent need for a more sensitive and accurate test to screen for the infection of *F. necrophorum*.

mNGS refers to high-throughput sequencing of the entire genome of organisms in a specimen, which is a new technology that can deeply and rapidly identify infectious pathogens (Bharucha et al., 2020). Studies have reported that the consistency between mNGS and blood culture results can reach 93.7%, which is more effective than other detection methods in the identification of pathogens causing sepsis (Timsit et al., 2020; Blauwkamp et al., 2019). In addition, the overall positive rate of mNGS sequencing is significantly higher than that of body fluid culture, which can identify potential bacterial pathogens missed by traditional culture methods (Hogan et al., 2021; Li et al., 2021). When interpreting mNGS results, we usually make a comprehensive judgment to distinguish true-positive and false-positive pathogens based on sequence number, coverage, abundance, and documentary evidence. The expert consensus recommended 20 million reads as average sequencing data for mNGS. In this sample, the amount of sequencing data was 45,185,658, which demonstrates that the data volume for mNGS sequencing was sufficient. However, the host abundance ratio achieved as high as 99.89%, leading to a low read number of microbes (<3k reads). Therefore, efficient host depletion is essential to mNGS. In this case, mNGS from the blood showed that the patient was infected with *F. necrophorum*, indicating moderate confidence. mNGS had further identified an array of additional pathogens. Significantly, *F. necrophorum* was recognized as the relative dominant species in the clearly defined species, followed by *Alishewanella aestuarii* and *Pseudoramibacter alactolyticus*. The entire microbial consortia promoted the development of sepsis. Throat swab culture results obtained 5 days after admission indicated *F. necrophorum* and *Streptococcus viridans*, which verified the accuracy of the mNGS results. *Streptococcus vermicularis*, as the most common colonizing bacteria in the human throat, was not considered a pathogenic bacterium here. In addition, the detection time of mNGS is shorter than that of culture, so infection can be confirmed as soon as possible and targeted medication can be directed. Currently, mNGS testing time can be cut down to 24 h. In addition to mNGS, 16S amplicon sequencing can quickly and efficiently identify bacteria as well, such as anaerobic bacteria. However, the species detected by 16S amplicon sequencing are mainly bacteria. The metabolic pathway, infection degree, virulence, and other properties of microorganisms cannot be detected by 16S amplicon sequencing. Therefore, in this case, since the pathogen type could not be determined, we could not only detect the bacteria by 16S amplicon sequencing and ignore other types of pathogens. Although reliable drug susceptibility information cannot be obtained, it should still be used as an important supplement to traditional pathogen detection, especially for such critical case as community-acquired infections (Timsit et al., 2020; Blauwkamp et al., 2019; Hogan et al., 2021; Li et al., 2021).

Early application of targeted antibiotics is a key factor affecting the prognosis of *F. necrophorum* infection. Antibiotic therapy with anaerobic coverage must be rapidly introduced (Nygren and Holm, 2020). As *F. necrophorum* contains β-lactamase, it is naturally resistant to quinolones and aminoglycosides, as well as having poor sensitivity to macrolides and tetracycline. Metronidazole is recommended in combination with β-lactam antibiotics (Sacco et al., 2019; Tay and Vasoo, 2022). In current research, the average duration of antibiotic use was as long as 2–4 weeks, and some severe patients were treated for 8–16 weeks (Sheehan et al., 2019; Gama et al., 2020; Tamura et al., 2020; El Chebib et al., 2020; Repper et al., 2020; Monroe and Amlie-Lefond, 2018; Dal Bo et al., 2019; Whittle et al., 2018; Rana et al., 2017; Sinatra and Alander, 2017; He et al., 2015; Creemers-Schild et al., 2014). In this case, to cover the anaerobic bacteria as well as some pathogenic or coinfected G-bacilli and streptococci, the broad-spectrum carbapenem antibiotics were initially used empirically. The use of mNGS to accurately detect pathogens at the early stage of the disease guided the transformation of the anti-infection program from empirical to target. Targeted use of metronidazole for anti-infection began before complications such as Lemierre’s syndrome appeared. This regimen, which was used early, shortened the course of anti-infective treatment and achieved good clinical efficacy, with no drug-related toxic reactions occurring. The hypercoagulable state of *F. necrophorum* infection leads to a high risk of thrombosis, and the timing of anticoagulant therapy is controversial (Lee et al., 2020; Carius et al., 2022). Recent studies suggested that early use of anticoagulants can help prevent the occurrence of disseminated intravascular coagulation (DIC) and Lemierre’s syndrome caused by sepsis (Tamura et al., 2020; Hayakawa et al., 2016). This patient was treated with low-molecular-weight heparin for anticoagulation in the early stage. There was no sign of jugular vein thrombosis or septic embolism in the long-term follow-up. More proactive anticoagulant therapy and individualized anticoagulant regimens may be considered for such infection, which can bring more prognostic benefits.

There are still some limitations to the case report. Rapid identification of the pathogen and its susceptibility to antibiotics are crucial steps in sepsis treatment. In this case, mNGS only detected *F. necrophorum*, but its drug resistance could not be determined. In addition, human infection with *F. necrophorum* usually involves *F. necrophorum* subsp. *funduliforme*, which we were unable to identify in this case. PCR has potential advantages in the identification of subspecies of *F. necrophorum.* In a study of adult patients with tonsillitis, Jensen et al. (2007) used a novel *F. necrophorum* subspecies-specific real-time PCR assay to compare the results and found that 51% of 105 patients with tonsillitis were positive for *F. necrophorum* subsp. *funduliforme*, compared with 21% of the control group. In future studies, we can pay more attention to the identification of resistance in mNGS to *F. necrophorum* and pathogenic subspecies. More similar cases can also be collected to explore the advantages and disadvantages of mNGS in the detection of *F. necrophorum*.

## Conclusion

*F. necrophorum* infection is rare in children, which can lead to serious sepsis and high mortality. Early treatment with targeted antibiotics is generally effective. The use of mNGS can deeply and rapidly identify infectious pathogens, which is more sensitive than the traditional culture method. It also has good clinical significance for the treatment of antibacterial drugs and can remarkably improve the survival rate of patients.

## Data availability statement

The datasets presented in this study can be found in online repositories. The names of the repository/repositories and accession number(s) can be found in the article/**Supplementary Material**.

## Ethics statement

The studies involving humans were approved by West China Second University Hospital, Sichuan University. The studies were conducted in accordance with the local legislation and institutional requirements. Written informed consent for participation in this study was provided by the participants’ legal guardians/next of kin. Written informed consent was obtained from the individual(s), and minor(s)’ legal guardian/next of kin, for the publication of any potentially identifiable images or data included in this article.

## Author contributions

LR summarized the case information and designed the manuscript. HZ drafted the manuscript. ZL analyzed the data. YG designed the bioinformatic analysis. DL and HL revised the manuscript. All authors contributed to the article and approved the submitted version.
