# Supplementary material for: Case report: Metagenomics next-generation sequencing in the diagnosis of septic shock due to Fusobacterium necrophorum in a 6-year-old child
Source: Front Cell Infect Microbiol. 2024 Feb 16;14:1236630. doi: 10.3389/fcimb.2024.1236630 (PMC10904578; doi:10.3389/fcimb.2024.1236630)
Supplement: Supplementary file 2 [file Table_1.docx]

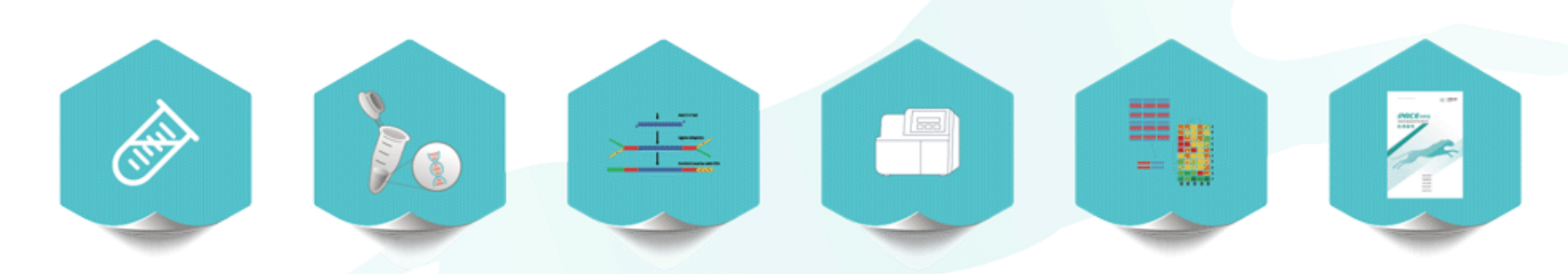


1. **Flow chart of mNGS detection process**

**Computer Sequencing**

**Result Presentation**

**Sample Collection**

**Nucleic Acid Extraction**

**Library Construction**

**Bioinformation Analysis**

1. **Sequencing and Quality Control Process of mNGS**

**Dry lab(informatics) pipeline**

**Wet lab pipeline**

- Low load extraction technology, significantly improve pathogen detection rate.
- Cell free nucleic acid as the detection object, more comprehensive and real coverage of DNA or RNA.

**Nucleic acid extraction**

**Library construction**

- Respiratory infections:BALF, airway aspirates, induced sputum, deep sputum, lung tissue, blood
- Central nervous system infections: Cerebrospinal fluid(CSF) , brain drainage fluid, blood
- Bloodstream infection: Blood
- Other infections: lesion site or blood, etc.

**Specimen collection**

**Data quality control**

- Number of specific sequences, qualitative and quantitative.
- confidence
- Pathogen pathogenicity
- Host immune status
- Accurate identification of bacteria, fungi and viruses.
- Analysis of drug resistance genes and virulence genes.
- Average amount of sequencing data 20M
- Q30 ≧ 90%

**Relative quantification of pathogens**

**Accurate interpretation and analysis**

**al analysis**

- Specific sequence number, precise qualitative, relative quantitative.
- Degree of confidence, multi-dimensional evaluation of the accuracy of the number of specific sequences.

**Report interpretation**

**Removal of human reads**

**Microbial identification**

**and Functional analysis**

Build human pan-genome database, remove the host sequence, eliminate human data interference.

- Illumina sequencing platform
- Sequencing read length ≧ 75bp

**Sequencing**
